# Supplementary material for: Therapeutic effect of and mechanisms underlying the effect of miR-195-5p on subarachnoid hemorrhage-induced vasospasm and brain injury in rats
Source: PeerJ. 2021 Jun 22;9:e11395. doi: 10.7717/peerj.11395 (PMC8231314; doi:10.7717/peerj.11395)
Supplement: Supplemental Information 1 [file peerj-09-11395-s001.pdf]

Supplement data

1. miR-195-5p and mimic: PhalanxBio Inc.
    - miR-195-5p conc. 5nmol/ml/kg

2. Antibody:

iNOS: BD biosciences, 610329
eNOS: BD biosciences, 610279
NF-κB:proteintech, 10745-1-AP
p-NF-κB: cell signaling, 3033
Beta-actin: Sigma, A5441

3. Image assay software: ImageJ, NIH.

4. Analysis software: SPSS

5. Target Site Prediction

The miR-195-5p sequence was obtained from miRBase database (http://www.mirbase.org/), and miR-195-5p targets were performed by screening the databases: miRDB (http://mirdb.org/index.html), microR.org (http://www.microrna.org/) and miRwalk (http://mirwalk.umm.uni-heidelberg.de/). Then, from the list of mRNAs predicted to be targeted by miR-195-5p, the interaction with IKKα was specifically analyzed.

```
961  5  '… ttatttTGCTGCTtgatgat…….3'                IKKα
               | | | | | | |
         3'      UAGCAGCACAGAAAUAUUGGC   5'      has-miR-195-5p
```

The data were expressed as mean ± standard error of the mean and illustrated using GraphPad Prism software (version 8.0; GraphPad, Inc., La Jolla, CA, USA). Differences between the experimental groups were analyzed by one-way ANOVA was utilized, followed by a Tukey's post hoc test. (IBM SPSS Statistics, version 24.0; IBM Inc., Armonk, New York, USA). A p-value of < 0.05 was considered significant. All statistical operations were performed using.

	Control	SAH only	SAH+NC-mimic	SAH+miR-195-5p
For Morphometric Analysis	6	6	6	6
For Western blotting	6	6	6	6
For Cell death assay	6	6	6	6
For ELISA assay	6	6	6	6
Each group	24	24	24	24
Total animals				96

Raw data

Fig. 1 Cross-section area of the basilar artery

	Control	SAH	SAH+NC-mimic	SAH+miR-195-5p
Cross-sectional area of basilar artery (μm2)	78169.26	45632.01	39912.12	78214.33
	72015.7	52604.98	38674.33	71234.23
	68101.45	41192.36	42126.32	82136.34
	79269.43	50741.77	41937.24	78479.26
	74318.79	43935.48	51312.2	79351.59
	75030.11	44713.69	*	75153.65
N	6	6	5	6

One-way anova

F(3,19)=102.623, p < 0.000

Tukey test

	contorl	SAH	SAH+NC-mimic	SAH+miR-195-5p
**contorl**		0.000	0.000	0.639
		***	***	
**SAH**			0.503	0.000
				###

Raw data
Fig. 2 western blotting for iNOS

	Control		SAH only		SAH+NC-mimic		SAH+miR-195-5p	
	% of control	iNOS/beta-actin	% of control	iNOS/beta-actin	% of control	iNOS/beta-actin	% of control	iNOS/beta-actin
	100.000	0.135	220.007	0.297	235.259	0.317	126.415	0.171
	100.000	0.116	218.828	0.255	191.859	0.223	93.0	0.108
	100.000	0.120	231.992	0.278	176.916	0.212	139.948	0.167
	100.000	0.071	187.598	0.134	205.860	0.147	131.051	0.093
	100.000	0.048	195.793	0.095	*	*	148.066	0.072
	100.000	0.144	*	*	*	*	*	*
N		6		5		4		5

One-way anova

F(3,16)=49.608, p < 0.000

Tukey test

	**contorl**	**SAH**	**SAH+NC-mimic**	**SAH+miR-195-5p**
**contorl**		0.000	0.000	0.084
		***	***	
**SAH**			0.893	0.000
				###

Raw data
Fig. 3 western blotting for eNOS

	Control		SAH only		SAH+NC-mimic		SAH+miR-195-5p	
	% of control	eNOS/beta-actin	% of control	eNOS/beta-actin	% of control	eNOS/beta-actin	% of control	eNOS/beta-actin
	100.000	0.144	83.050	0.120	74.955	0.108	116.517	0.168
	100.000	0.125	87.862	0.110	93.721	0.117	101.254	0.126
	100.000	0.128	93.840	0.120	77.441	0.099	110.166	0.141
	100.000	0.076	89.418	0.068	86.113	0.066	111.698	0.085
	100.000	0.052	74.376	0.039	*	*	98.728	0.051
	100.000	0.154	*	*	*	*	*	*
N		6		5		4		5

One-way anova

$F(3,16)=15.748, p < 0.000$

Tukey test

	contorl	SAH	SAH+NC-mimic	SAH+miR-195-5p
**contorl**		0.010	0.004	0.240
		*	**	
**SAH**			0.926	0.000
				###

Raw data

Fig. 4 western blotting for p-NF-κB and NF-κB

	Control		SAH only		SAH+NC-mimic		SAH+miR-195-5p	
	% of control	p-NF-κB/NF-κB	% of control	p-NF-κB/NF-κB	% of control	p-NF-κB/NF-κB	% of control	p-NF-κB/NF-κB
	100.000	0.172	145.502	0.250	147.378	0.254	83.909	0.144
	100.000	0.149	155.807	0.231	150.620	0.224	76.956	0.114
	100.000	0.153	161.155	0.246	180.048	0.275	84.268	0.129
	100.000	0.091	116.792	0.106	119.784	0.109	103.744	0.094
	100.000	0.062	197.345	0.122	199.204	0.123	90.423	0.056
	100.000	0.184	*	*	*	*	*	*
N		6		5		5		5

One-way anova

$F(3,17)=15.796$, $p < 0.000$

Tukey test

	contorl	SAH	SAH+NC-mimic	SAH+miR-195-5p
contorl		0.002	0.001	0.779
		**	**	
SAH			0.990	0.001
				##

Raw data
Fig. 5 western blotting for p-IκB-α

	Control		SAH only		SAH+NC-mimic		SAH+miR-195-5p	
	% of control	p-IκB-α/beta-actin	% of control	p-IκB-α/beta-actin	% of control	p-IκB-α/beta-actin	% of control	p-IκB-α/beta-actin
	100.000	0.160	757.094	1.209	873.106	1.394	431.706	0.689
	100.000	0.138	613.077	0.845	594.407	0.819	369.400	0.509
	100.000	0.142	1194.371	1.692	1254.171	1.776	517.871	0.734
	100.000	0.084	1097.518	0.926	1097.737	0.926	407.476	0.344
	*	*	*	*	*	*	*	*
	*	*	*	*	*	*	*	*
N		4		4		4		4

One-way anova

$F(3,12)=16.590, p < 0.000$

Tukey test

	contorl	SAH	SAH+NC-mimic	SAH+miR-195-5p
**contorl**		0.000	0.000	0.145
		***	***	
**SAH**			0.992	0.024
				#

Supplement data

Fig. 2 iNOS

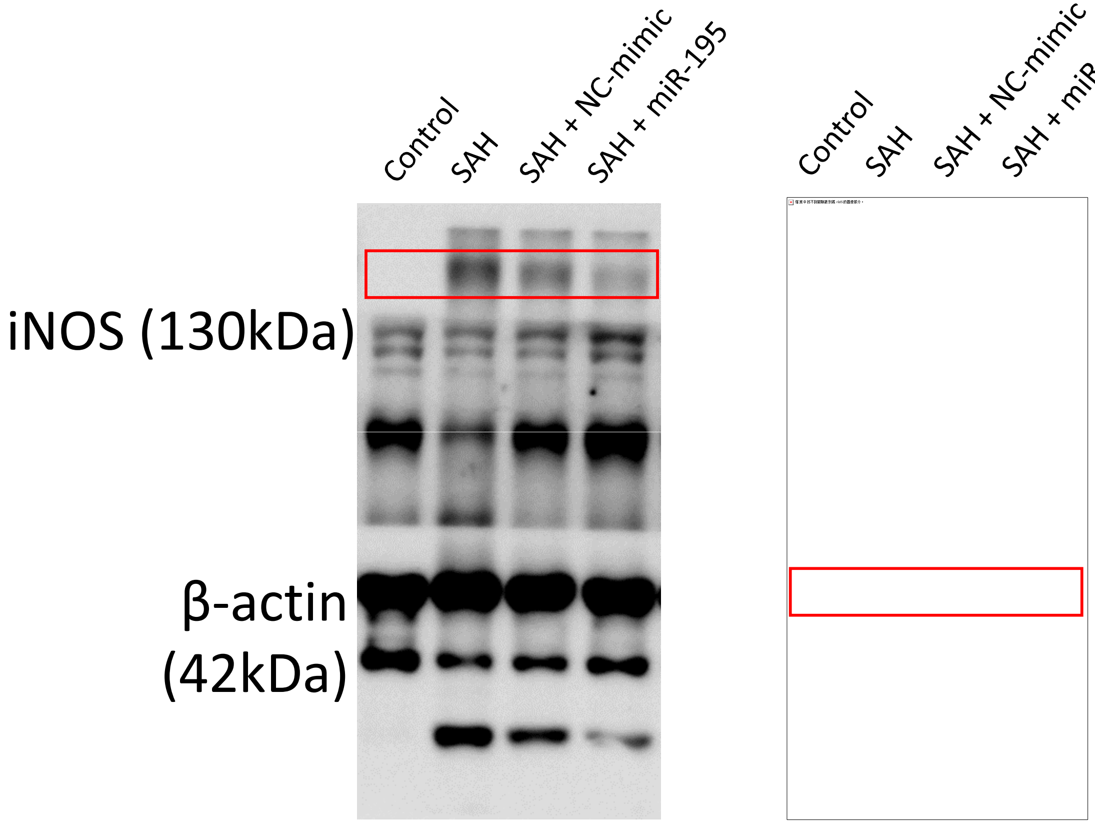

Fig. 3 eNOS

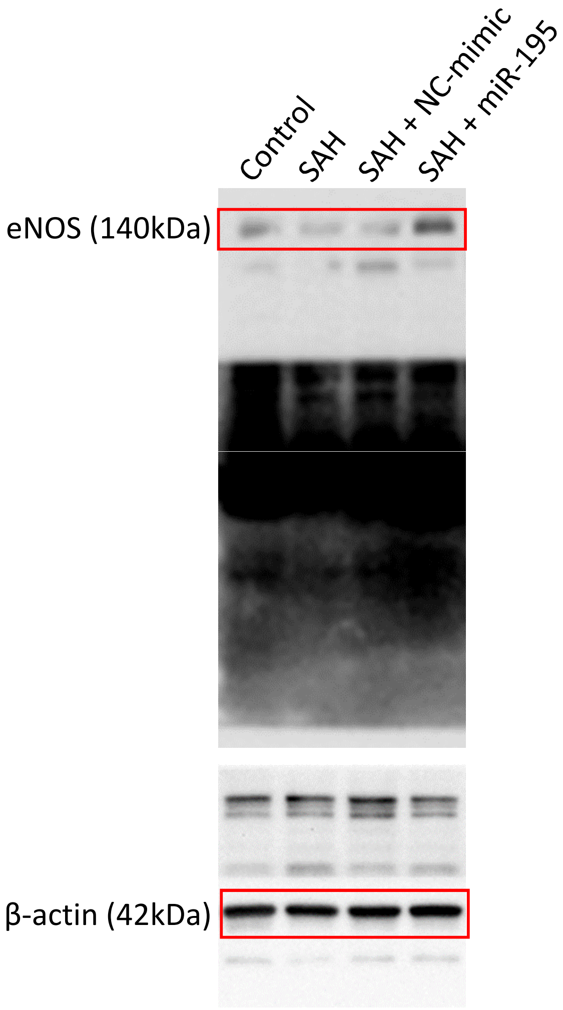

Supplement data

Fig. 4 p-NF-κB, NF-κB

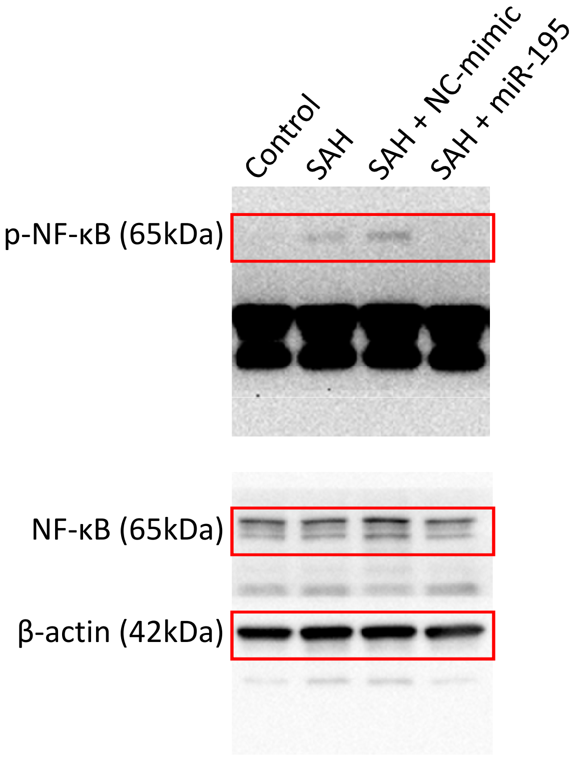

Fig. 5 p-IκB-α

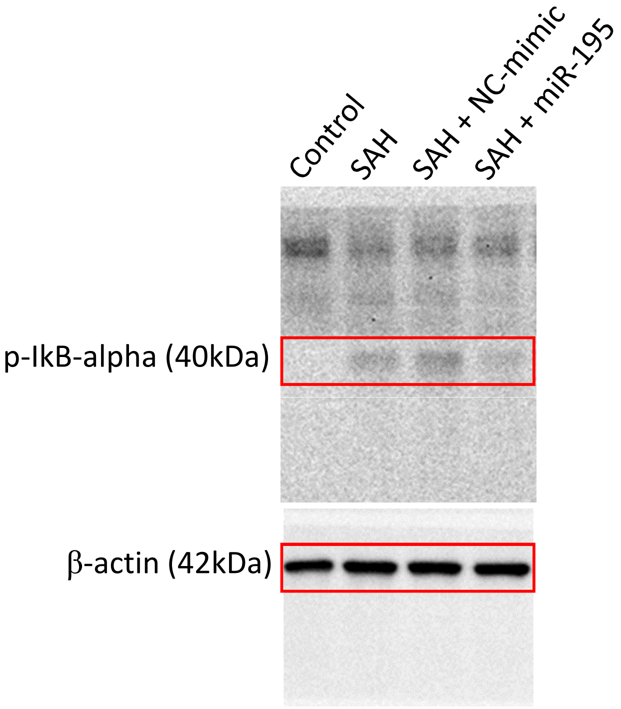

Raw data: Fig. 6(a) cell death assay for brain tissue

**Cortex**	Control		SAH only		SAH+NC-mimic		SAH+miR-195-5p	
	O.D.	% of control	O.D.	% of control	O.D.	% of control	O.D.	% of control
	0.055	103.6	0.048	90.4	0.054	102.5	0.056	105.6
	0.052	98.5	0.049	92.4	0.053	99.5	0.053	100.5
	0.058	110.7	0.047	89.3	0.047	88.3	0.055	103.6
	0.049	92.4	0.055	103.6	0.047	89.3	0.046	87.3
	0.048	91.4	0.048	91.4	0.05	95.4	0.052	97.5
	0.055	103.6	0.048	90.4	0.055	103.6	0.05	95.4
N		6		6		6		6

One-way anova

F(3,20)=1.315, $p < 0.297$

Tukey test

	contorl	SAH	SAH+NC-mimi	SAH+miR-195-5p
**contorl**		0.263	0.775	0.968
**SAH**			0.787	0.493

Raw data: Fig. 6(a) cell death assay for brain tissue

Hippcampus	Control		SAH only		SAH+NC-mimic		SAH+miR-195-5p	
	O.D.	% of control	O.D.	% of control	O.D.	% of control	O.D.	% of control
	0.061	109.6	0.059	105.5	0.054	96.2	0.06	107.5
	0.054	96.2	0.055	98.3	0.051	91.1	0.056	100.3
	0.058	104.4	0.06	106.5	0.059	105.5	0.062	111.6
	0.057	102.4	0.057	101.4	0.054	96.2	0.054	96.2
	0.05	89.1	0.054	97.3	0.051	91.1	0.056	100.3
	0.055	98.3	0.053	95.2	0.06	107.5	0.059	105.5
N		6		6		6		6

One-way anova

F(3,20)=0.850, p < 0.483

Tukey test

	contorl	SAH	SAH+NC-mimi	SAH+miR-195-5p
contorl		0.997	0.937	0.752
SAH			0.865	0.852

Raw data: Fig. 6(a) cell death assay for brain tissue

Dentate gyrus	Control		SAH only		SAH+NC-mimic		SAH+miR-195-5p	
	O.D.	% of control	O.D.	% of control	O.D.	% of control	O.D.	% of control
	0.06	106.8	0.115	206.5	0.125	224	0.082	146.9
	0.056	99.7	0.108	194.2	0.113	202.4	0.069	124.3
	0.053	95.5	0.12	216	0.105	189	0.1	178.8
	0.053	94.5	0.102	183.9	0.086	154	0.08	142.8
	0.06	106.8	0.083	149	0.118	211.6	0.074	132.5
	0.054	96.6	*	*	*	*	*	*
N		6		5		5		5

One-way anova

$F(3,17)=25.264, p < 0.000$

Tukey test

	contorl	SAH	SAH+NC-mimi	SAH+miR-195-5p
**contorl**		0.000	0.000	0.012
		***	***	*
**SAH**			0.964	0.017
				#

Raw data: Fig. 6(b) ELISA data of TNF-α

Cortex	control	SAH only	SAH+NC-mimic	SAH+miR-195-5p
TNF-α Conc. (pg/ml)	86.25	64.97	*	69.36
	75.79	*	84.23	87.11
	64.69	82.67	82.78	69.42
	54.74	91.89	71.24	*
	72.63	94.18	89.98	73.99
	52.43	87.69	82.36	87.15
N	6	5	5	5

One-way anova

F(3,17)=2.700, p < 0.078

Tukey test

	**contorl**	**SAH**	**SAH+NC-mimic**	**SAH+miR-195-5p**
**contorl**		0.083	0.152	0.455
**SAH**			0.988	0.736

Raw data: Fig. 6(b) ELISA data of TNF-α

Hippocampus	control	SAH only	SAH+NC-mimic	SAH+miR-195-5p
TNF-α Conc. (pg/ml)	93.36	98.28	116.68	*
	87.44	84.31	97.28	76.27
	76.21	76.16	*	67.06
	69.11	97.4	109.42	84.61
	72.36	83.13	94.87	86.24
	54.46	103.62	67.94	98.41
N	6	6	5	5

One-way anova

$F(3,18)=2.548, p < 0.088$

Tukey test

	**contorl**	**SAH**	**SAH+NC-mimic**	**SAH+miR-195-5p**
**contorl**		0.275	0.080	0.837
**SAH**			0.852	0.780

Raw data: Fig. 6(b) ELISA data of TNF-α

Dentate Gyrus	control	SAH only	SAH+NC-mimic	SAH+miR-195-5p
TNF-α Conc. (pg/ml)	75.36	107.48	103.36	76.44
	61.36	123.36	106.35	*
	64.14	114.21	99.51	74.94
	74.36	108.49	87.03	69.17
	73.32	*	126.37	59.1
	*	87.36	*	89.65
N	5	5	5	5

One-way anova

$F(3,16)=14.816, p < 0.000$

Tukey test

	contorl	SAH	SAH+NC-mimic	SAH+miR-195-5p
**contorl**		0.000	0.001	0.942
		***	**	
**SAH**			0.959	0.001
				##
